# Multiparametric MRI subregion radiomics for preoperative assessment of high-risk subregions in microsatellite instability of rectal cancer patients: a multicenter study

**DOI:** 10.1097/JS9.0000000000001335

**Published:** 2024-03-18

**Authors:** Zhiping Cai, Zhenyu Xu, Yifan Chen, Rong Zhang, Baoliang Guo, Haixiong Chen, Fusheng Ouyang, Xinjie Chen, Xiaobo Chen, Dechao Liu, Chun Luo, Xiaohong Li, Wei Liu, Cuiru Zhou, Xinqun Guan, Ziwei Liu, Hai Zhao, Qiugen Hu

**Affiliations:** a Department of Radiology, Shunde Hospital, Southern Medical University (The First People’s Hospital of Shunde); b Department of Radiology, The First People’s Hospital of Foshan, Foshan; c Department of Radiology, Guangdong Provincial People’s Hospital (Guangdong Academy of Medical Sciences), Southern Medical University; d Guangdong Provincial Key Laboratory of Artificial Intelligence in Medical Image Analysis and Application, Guangzhou, People’s Republic of China

**Keywords:** microsatellite instability, multiparametric magnetic resonance imaging, radiomics, rectal cancer, subregion

## Abstract

**Background:**

Microsatellite instability (MSI) is associated with treatment response and prognosis in patients with rectal cancer (RC). However, intratumoral heterogeneity limits MSI testing in patients with RC. The authors developed a subregion radiomics model based on multiparametric MRI to preoperatively assess high-risk subregions with MSI and predict the MSI status of patients with RC.

**Methods:**

This retrospective study included 475 patients (training cohort, 382; external test cohort, 93) with RC from two participating hospitals between April 2017 and June 2023. In the training cohort, subregion radiomic features were extracted from multiparametric MRI, which included T2-weighted, T1-weighted, diffusion-weighted, and contrast-enhanced T1-weighted imaging. MSI-related subregion radiomic features, classical radiomic features, and clinicoradiological variables were gathered to build five predictive models using logistic regression. Kaplan–Meier survival analysis was conducted to explore the prognostic information.

**Results:**

Among the 475 patients [median age, 64 years (interquartile range, IQR: 55–70 years); 304 men and 171 women], the prevalence of MSI was 11.16% (53/475). The subregion radiomics model outperformed the classical radiomics and clinicoradiological models in both training [area under the curve (AUC)=0.86, 0.72, and 0.59, respectively] and external test cohorts (AUC=0.83, 0.73, and 0.62, respectively). The subregion-clinicoradiological model combining clinicoradiological variables and subregion radiomic features performed the optimal, with AUCs of 0.87 and 0.85 in the training and external test cohorts, respectively. The 3-year disease-free survival rate of MSI groups predicted based on the model was higher than that of the predicted microsatellite stability groups in both patient cohorts (training, *P*=0.032; external test, *P*=0.046).

**Conclusions:**

The authors developed and validated a model based on subregion radiomic features of multiparametric MRI to evaluate high-risk subregions with MSI and predict the MSI status of RC preoperatively, which may assist in individualized treatment decisions and positioning for biopsy.

## Introduction

Highlights

- This study was conducted on 475 patients with rectal cancer (RC) to preoperatively assess high-risk subregions with microsatellite instability (MSI) and predict the MSI status of RC patients, based on multiparametric MRI.
- The subregion-clinicoradiological model combining clinicoradiological variables and subregion radiomic features demonstrating favorable performance and prognostic stratification value.
- The subregion radiomics model can reveal where high-risk subregions of MSI be located and potentially assist to MSI testing.

Colorectal cancer is globally prevalent and ranks third in both incidence and mortality rates among all cancers. Rectal cancer (RC) accounts for ~31% of all colorectal cancer cases^1^ and is caused by genetic expression changes, of which 10–20% can be detected based on a hypermutable phenotype called microsatellite instability (MSI)^2^. MSI is characterized by defects in the mismatch repair (MMR) system, and numerous studies have confirmed its association with Lynch syndrome^1,3^. Patients with RC and MSI may exhibit resistance to 5-FU-based chemotherapy and are more likely to benefit from immunotherapy^4–6^. These studies indicate that MSI status is closely related to the treatment response and prognosis of patients with RC^7–9^.

The National Comprehensive Cancer Network (NCCN) and the European Society for Medical Oncology (ESMO) guidelines recommend MSI testing for all patients with RC^10,11^. MSI can be detected by polymerase chain reaction amplification of specific microsatellite markers or by observing the loss of expression of an MMR protein using immunohistochemistry^2^. However, intratumoral heterogeneity poses limitations to MSI testing in patients with RC^12,13^. A noninvasive method is needed to preoperatively assess high-risk subregions with MSI and predict the MSI status of patients with RC. These findings have significant implications for pathological sampling, treatment response, and prognosis.

MRI is extensively used to diagnose, stage, assess prognosis, and track treatment response in RC^14–16^. Radiomics can convert digital images into quantitative features to capture detailed information of tumors^17^. MRI-based radiomics is an effective analytical method for predicting MSI status in RC^18,19^. However, the analyses in these studies were limited to the whole tumor, and no further analyses were performed for the subregions. To our knowledge, the use of subregion radiomic features of multiparametric MRI to analyze the relationship between subregions and MSI in RC has not been well established.

Therefore, this study aimed to develop a subregion radiomics model based on multiparametric MRI to preoperatively assess high-risk subregions with MSI and to predict the MSI status of patients with RC. The findings may aid in making clinical decisions regarding personalized treatment for patients with RC and the positioning for retrieving pathological materials.

## Materials and methods

### Participants

This retrospective multicenter study was approved by the Institutional Ethics Review Board of Shunde Hospital of Southern Medical University, which waived the requirement for informed consent. The training cohort was recruited from The First People’s Hospital of Foshan (Center A), and the external test cohort patients were recruited from The First People's Hospital of Shunde (Center B). This study was conducted in accordance with the REMARK criteria^20^ (Supplemental Digital Content 1, http://links.lww.com/JS9/C194).

**Figure 1:**
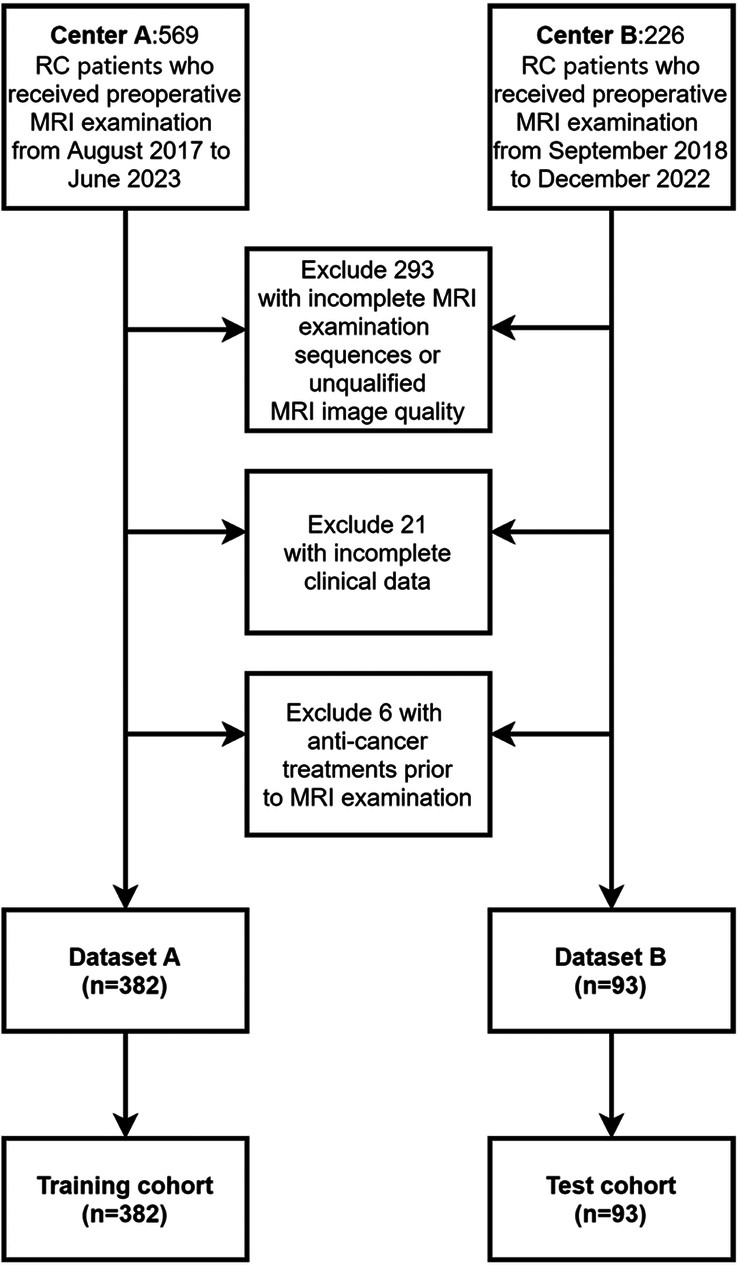
Flowchart of inclusion and exclusion criteria for eligible patients in the study. RC, rectal cancer.

The data used in this study included preoperative MRI findings and the clinical data of patients diagnosed with RC through pathological examination at the two participating hospitals between April 2017 and June 2023. The inclusion criteria were as follows: (A) histopathological diagnosis of RC, (B) less than 1 week between MRI examination and surgery, and (C) available MMR status confirmed by immunohistochemical staining. The exclusion criteria were as follows: (A) incomplete MRI examination sequences or unqualified MRI image quality, (B) incomplete clinical data, and (C) anticancer treatment prior to MRI examination. The training cohort comprised 382 patients from Center A, whereas the external test cohort comprised 93 patients from Center B. The inclusion and exclusion processes are illustrated in Figure 1.

### Histopathological examination

Histological slices from the two centers were evaluated by two experienced pathologists from Center B using the same criteria, without access to the patients’ clinical data. In case of inconsistencies, a third senior pathologist was consulted.

Following the research recommendations of Luchini *et al*.^8^, MSI status was divided into MSI (MSI-High) and microsatellite stability (MSS, including MS-Low and MS-Stable). MSI status was assessed by detecting the expression of MMR proteins (MLH1, MSH2, MSH6, and PMS2) using immunohistochemical staining. Patients with RC were classified into two groups: the MSI group (negative staining observed for any one of the MMR proteins) and the MSS group (positive staining for all four MMR proteins)^2^.

### Clinicoradiological data and follow-up

Clinical data, including age, sex, carcinoembryonic antigen (CEA) levels (considered abnormal if greater than 5 ug/l), and carbohydrate antigen 19-9 (CA19-9) levels (considered abnormal if greater than 37 U/ml), were extracted from the medical record archives of the participating centers.

Radiologists A and B (with 5 and 10 years of experience in abdominal imaging diagnosis, respectively), unaware of the patients’ MSI status, assessed radiological data, including tumor location, tumor (T) stage, and lymph node (N) stage. Tumor location was defined based on the distance from the inferior part of the tumor to the anal verge as low (<5 cm), middle (5–10 cm), or high (>10 cm)^21^. Radiological TN stage was determined based on the 8th edition of the American Joint Committee on Cancer (AJCC) staging system. The final radiological data were based on the observations made by the more experienced radiologist B. Inter-reader variation of radiological features was measured with κ-statistic (κ > 0.75, excellent agreement; 0.40 ≤ κ ≤ 0.75, good agreement; κ<0.40, poor agreement).

All patients underwent postoperative follow-up using abdominal ultrasound (US), contrast-enhanced computed tomography (CT), or MRI. Tumor recurrence or metastasis was defined based on typical imaging features observed on CT, MRI, or US or on pathological results. Disease-free survival (DFS) was defined as the time from the date of surgery to the date of first recurrence, metastasis, or last follow-up. The follow-up period was 23 October 2023.

### MRI protocol and image preprocessing

Preoperative examinations of patients were performed at both the centers using one of the five available MRI scanners. The imaging sequences included T2-weighted imaging (T2WI), T1-weighted imaging (T1WI), diffusion-weighted imaging (DWI), and contrast-enhanced T1-weighted imaging (CE-T1WI). The imaging protocol parameters are listed in Supplementary Table 1 (Supplemental Digital Content 2, http://links.lww.com/JS9/C195).

The MRI images of all patients were preprocessed as given below to eliminate heterogeneity between images and improve the reproducibility and comparability of the results^22^. First, the ANTs software (version 2.5.0; https://github.com/ANTsX/ANTs) was used to spatially align the four MRI sequences, ensuring voxel-level spatial consistency of the tumor between the four sequences. Second, an N4 bias field correction was performed on the images to remove bias field artifacts^23^. Third, the intensity values of the images were standardized by setting the mean to zero and the variance to 1^24^. Finally, the images were resampled using B-spline interpolation with a voxel spacing of 1×1×1 mm^3^ to standardize the voxel spacing. The gray level of the image was discretized and normalized to the order of 32^25^.

### Tumor segmentation and subregion label map generation

The volume of interest of the tumor was manually segmented by radiologists A and B using 3D Slicer software (version 5.2.2; https://www.slicer.org). The following steps were used to generate the subregion label map.

First, four MRI sequences were laterally merged to obtain a multidimensional sequence. The tumor was then clustered into 10 subregions using the fuzzy c-means (FCM) algorithm^26^ based on the voxel value^26^. This resulted in a new label map with ten subregions, which was used to preliminarily extract the radiomic features of the subregions for further clustering (hereafter referred to as secondary radiomic features).

Next, the secondary radiomics features (used for clustering, including 18 first-order statistical features and 74 texture features)^27,28^ were extracted from the subregions of the four MRI sequences, resulting in 368 secondary radiomic features. To obtain the optimal distribution and number of subregions, principal component analysis (PCA)^29^ was used to reduce the dimensionality of the secondary radiomic features, and the information retained after dimensionality reduction accounted for 0.85 of the original information. The Bayesian Gaussian mixed-model algorithm was then applied to cluster subregions with similar secondary radiomic features, with the number of subregions ranging from 1 to 10^30,31^. This resulted in a subregion-based label map with an optimal distribution and number of subregions for each patient.

**Figure 2:**
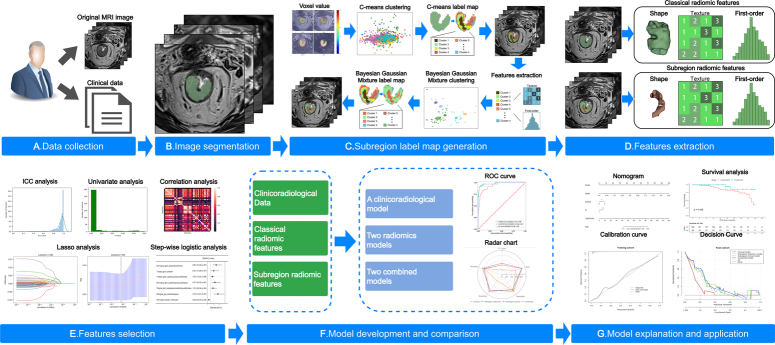
Flowchart of radiomics analysis in this study. (A) Collection of clinical data and multiparametric MRI images. (B) Tumor segmentation on the images. (C) Fuzzy c-means (FCM) and Bayesian gaussian mixed model clustering algorithms were used to generated subregion label map. (D) Extraction of classical radiomic features and subregion radiomic features. (E) A series of feature selection strategies were used to select MSI-related features. (F) The construction of models and the comparison between different models. (G) Application, validation, and survival prognosis analysis of the model.

Finally, a subregion-based label map was used to extract the subregion radiomic features, and the MSI status of the tumor subregions was consistent with that of the whole tumor (Fig. 2C).

### Subregion and classical radiomic feature extraction and selection

The pyradiomics software package (version 3.1.0; https://pyradiomics.readthedocs.io/en/latest) was used for feature extraction. The subregion radiomic features, including 18 first-order statistical features, 14 shape-based features, and 74 texture-based features were extracted using a subregion-based label map from four sequences^27,28^. A total of 424 subregional radiomic features were extracted from each subregion. Similarly, 424 classical radiomic features were extracted using a tumor-based label map. The z-scores were then used to normalize all radiomic features. The radiomics analysis process is shown in Figure 2.

Intraclass correlation coefficients (ICCs) were used to assess the reliability of radiomic features. The data of 30 patients were randomly selected for segmentation and feature extraction. Segmentation and feature extraction were performed by radiologists A and B, respectively, to assess interobserver reliability. Six months later, the data of the same 30 patients were segmented again by radiologist A to assess intraobserver reliability^32,33^. Only radiomic features with good interobserver and intraobserver reliability (both ICCs >0.75) were included in subsequent analyses. The results of the ICC analysis were shared by both classical and subregion radiomic features; however, the following steps were performed separately: Univariate logistic regression was used to select the radiomic features associated with MSI. Subsequently, Pearson’s correlation analysis was used to eliminate redundant features. If the correlation coefficient between two features was greater than 0.75, one of the features with a higher correlation with the other features was removed. Next, the least absolute shrinkage and selection operator (LASSO) algorithm^34^ was used to select the optimal subset, with penalty parameter tuning conducted through a 10-fold cross-validation. Finally, stepwise logistic regression with Akaike information criterion was used for feature selection.

### Clinicoradiological variable selection

In the training cohort, univariate logistic regression was used to analyze the association between clinicoradiological variables and MSI. Features with *P*<0.1 were selected for multivariable analysis using stepwise logistic selection with the Akaike information criterion. Thereafter, features with *P*<0.05 were chosen for model development.

### Model development

Several classification models were constructed using logistic regression (version 1.2.1; Scikit-learn) in the Python package based on the selected classical radiomic features, subregion radiomic features, and clinicoradiological variables. To avoid overfitting and train a robust model, a 10-fold cross-validation was performed in the training cohort to select the optimal parameters for the classification model. During training, the logistic regression model utilized the ‘elastic network’ regularization parameter (combining both L1 and L2 penalties). The grid search method was employed to determine the optimal parameters, including l1_ratio (which controls the balance of L1 and L2 regularization), C (the inverse of the regularization intensity), max_iter (the maximum number of iterations required for solver convergence), and tol (stop standard tolerance)^35^. The class_weight parameter was set to ‘balanced’ to mitigate model bias towards the majority class during training^35,36^. The overall predictive performance of the model was assessed by calculating the average area under the curve (AUC) of the validation cohort data across all folds. Subsequently, a logistic regression model was built for the training cohort using the optimal parameters and applied to all patients in the test cohort to assess the generalizability of the model.

The optimal number of subregions in each patient with RC varies, which is not conducive to constructing a joint model. Therefore, the following formula was used to calculate the subregion rad-score for each patient:


$${\mathrm{Rad}-\mathrm{score}}_{\mathrm{subregion}}=\sum_{i=1}^{V}\frac{{\mathrm{Rad}-\mathrm{score}}_{i}}{V}$$

Here, V represents the optimal number of subregions in patients with RC, and ${\mathrm{Rad}-\mathrm{score}}_{i}$ denotes the rad-score of each subregion. The higher the risk of MSI in a subregion, the higher the ${\mathrm{Rad}-\mathrm{score}}_{i}$ . Similarly, the higher the number of high-risk subregions with MSI in an RC tumor, the higher the ${\mathrm{Rad}-\mathrm{score}}_{\mathrm{subregion}}$ .

Based on the selected classical radiomic features, subregion radiomic features, and clinicoradiological features, five models were trained: the clinicoradiological model (clinicoradiological variables), subregion radiomics model (subregion radiomic features), classical radiomics model (classical radiomic features), subregion-clinicoradiological model (subregion radiomic features, clinicoradiological features), and combined model (subregion radiomic features, classical radiomic features, and clinicoradiological variables).

### Statistical analysis

Continuous variables were described using median and interquartile range (IQR), whereas categorical variables were described using frequencies and percentages. Logistic regression analyses, including both univariate and multivariate models, were conducted to assess the association between various patient characteristics and the odds of MSI. The DeLong test was used to compare the AUCs of the different classification models. Decision curve analysis was performed to compare the differences in net benefits between the different models at the threshold probability. Five indicators–AUC, F1_score, accuracy, sensitivity, and specificity–were employed to assess the performance of the prediction model. The optimal model was selected based on these indicators, and the goodness of fit of the optimal model was evaluated using the Hosmer–Lemeshow test. To assess the prognostic stratification value of the optimal model, patients were stratified into predicted MSI and MSS groups by maximizing the Youden index. Survival curves generated using the Kaplan–Meier method were compared using the log-rank test. Statistical analyses were performed using Python (version 3.8.16; https://www.python.org) and R (version 4.3.1; https://www.r-project.org). Statistical significance was considered at two-sided *P*<0.05, except for the univariate analysis of clinicoradiological variables, where *P*<0.1 was deemed significant.

## Result

### Patient characteristics

A total of 475 patients with RC were ultimately included in the study [median age, 64 years (IQR, 55–70 years); 304 men and 171 women]. The prevalence of MSI was 11.16% (53/475). Of the 475 patients, 282 (59%) were successfully followed up and included in the DFS analysis [median time, 18 (IQR, 7–31 months)]. The training cohort comprised 382 patients (80%) from Center A with a median age of 64 years (IQR, 50–70 years), among which 246 (64%) and 136 (36%) patients were male and female, respectively. The external test cohort consisted of 93 patients (20%) from Center B, with a median age of 63 years (IQR, 53–69 years). Of the external test cohort, 58 (62%) were male, and 35 (38%) were female. Additional details are presented in Table 1. Radiologists A and B showed consistent analysis of radiological features, as the kappa values were all > 0.750 (0.770–0.981, *P*<0.001, Supplementary Table 2, Supplemental Digital Content 2, http://links.lww.com/JS9/C195).

**Table 1:** Clinicoradiological characteristics of 475 patients with RC in different cohorts.

	Training cohort		External test cohort	
Characteristics	MSS (*N*=348)	MSI (*N*=34)	MSS (*N*=74)	MSI (*N*=19)
Clinical characteristics				
Age				
Median (IQR)	64.0 (55.0–70.0)	65.0 (53.3–69.0)	64.0 (55.0–69.0)	59.0 (44.5–67.0)
Sex				
Male	222 (63.8%)	24 (70.6%)	43 (58.1%)	15 (78.9%)
Female	126 (36.2%)	10 (29.4%)	31 (41.9%)	4 (21.1%)
CEA (μg/l)				
≤5 ug/l	195 (56.0%)	19 (55.9%)	48 (64.9%)	10 (52.6%)
>5 ug/l	153 (44.0%)	15 (44.1%)	26 (35.1%)	9 (47.4%)
CA19-9(U/ml)				
≤37 U/ml	301 (86.5%)	32 (94.1%)	66 (89.2%)	15 (78.9%)
>37 U/ml	47 (13.5%)	2 (5.9%)	8 (10.8%)	4 (21.1%)
Radiological characteristics				
Tumor location				
Low	158 (45.4%)	11 (32.4%)	41 (55.4%)	7 (36.8%)
Middle	126 (36.2%)	12 (35.3%)	30 (40.5%)	9 (47.4%)
High	64 (18.4%)	11 (32.4%)	3 (4.1%)	3 (15.8%)
T stage				
T1–T2	106 (30.5%)	9 (26.5%)	25 (33.8%)	3 (15.8%)
T3–T4	242 (69.5%)	25 (73.5%)	49 (66.2%)	16 (84.2%)
N stage				
N0	225 (64.7%)	27 (79.4%)	49 (66.2%)	10 (52.6%)
N1–N2	123 (35.3%)	7 (20.6%)	25 (33.8%)	9 (47.4%)

CA19-9, carbohydrate antigen 19-9; CEA, carcinoembryonic antigen; IQR, interquartile range; MSI, microsatellite instability; MSS, microsatellite stability; RC, rectal cancer.

### Clinicoradiological model

In the training cohort, univariate analysis revealed a significant association of tumor location and N stage with MSI. After multivariate analysis, the clinicoradiological model included tumor location [high vs. low; odds ratio (OR)=3.02 (95% CI: 1.22–7.48), *P*=0.017] and N stage [N1–N2 vs. N0; OR=0.39 (95% CI: 0.16–0.94); *P*=0.037]. The AUC of the clinicoradiological model for MSI prediction was 0.59 (95% CI: 0.49–0.69) in the training cohort and 0.62 (95% CI: 0.48–0.75) in the external test cohort (Supplementary Table 3, Supplemental Digital Content 2, http://links.lww.com/JS9/C195).

### Subregion and classical radiomics model

The median ICCs of the 424 radiomic features for interobserver and intraobserver agreement assessments were 0.971 (IQR, 0.923–0.991) and 0.929 (IQR, 0.834–0.964), respectively, suggesting marked reliability of the radiomic features. The complete radiomic feature selection process can be found in the Supplementary Material (Supplementary Table 4, Supplemental Digital Content 2, http://links.lww.com/JS9/C195).

**Figure 3:**
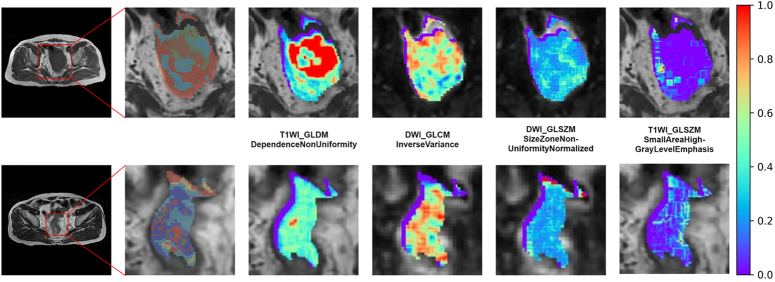
Example feature maps of the top four subregion radiomics features in a 67-year-old man with microsatellite instability (MSI) rectal cancer (RC) (top row) and a 65-year-old man with microsatellite stability (MSS) RC (bottom row). These images use colors to represent normalized feature values, with bluish colors indicating lower values and reddish colors indicating higher values. The subregions with high-risk of MSI showed higher values on GLDM_DependenceNonUniformity for T1 weighted imaging, CLCM_InverseVariance and GLSZM_SizeZoneNonUniformityNormalized for Diffusion-Weighted Imaging, and lower values for GLSZM_SmallAreaHighGrayLevelEmphasis at T1 weighted imaging.

Following feature dimensionality reduction, two radiomic models were constructed using the seven most significant subregion radiomic features or the six most significant classical radiomic features. The subregion radiomics features comprised three features on DWI, two features on T1WI, one feature on T2WI, and one feature on CE-T1WI. Similarly, the classical radiomic features consisted of four DWI, one T1WI, and one CE-T1WI features (Supplementary Figure 1, Supplemental Digital Content 2, http://links.lww.com/JS9/C195). In the training cohort, the subregion radiomics model predicted MSI with an AUC of 0.86 (95% CI: 0.79–0.93), whereas in the external test cohort, the AUC was 0.83 (95% CI: 0.74–0.92). In comparison, the classical radiomics model predicted MSI with an AUC of 0.72 (95% CI: 0.63–0.82) in the training cohort and 0.73 (95% CI: 0.60–0.85) in the external test cohort. The subregion radiomics model outperformed the classical radiomics model in both training (*P*=0.001) and external test cohorts (*P*=0.049). Therefore, the subregion radiomics model was selected as the primary radiomics model for subsequent analyses. The importance of subregion radiomic features is illustrated in Supplementary Figure 2 (Supplemental Digital Content 2, http://links.lww.com/JS9/C195), and the rad-score formula for each subregion is provided in the Supplementary Methods (Supplemental Digital Content 2, http://links.lww.com/JS9/C195). The top four subregion radiomic features identified based on the rank of absolute values of coefficients are visualized in Figure 3, which were associated with MSI.

### Comparison and validation of prediction models

**Figure 4:**
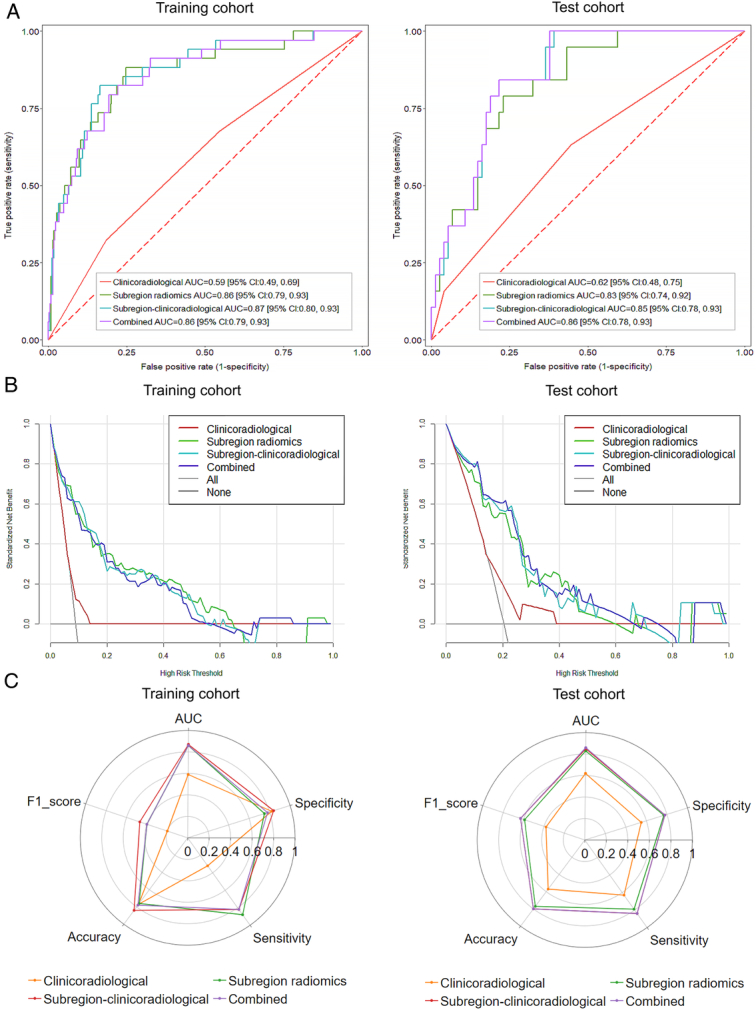
Overall comparison of the different models in the training and external test cohorts. (A) Receiver operating characteristic (ROC) curves, AUCs and 95% confidence intervals of different models in the training and external test cohorts. (B) Decision curves for predict MSI using different models. (C) Radar charts of different models using five metrics, subregion-clinicoradiological in the external test cohort overlaps most of the graphs with combined.

**Figure 5:**
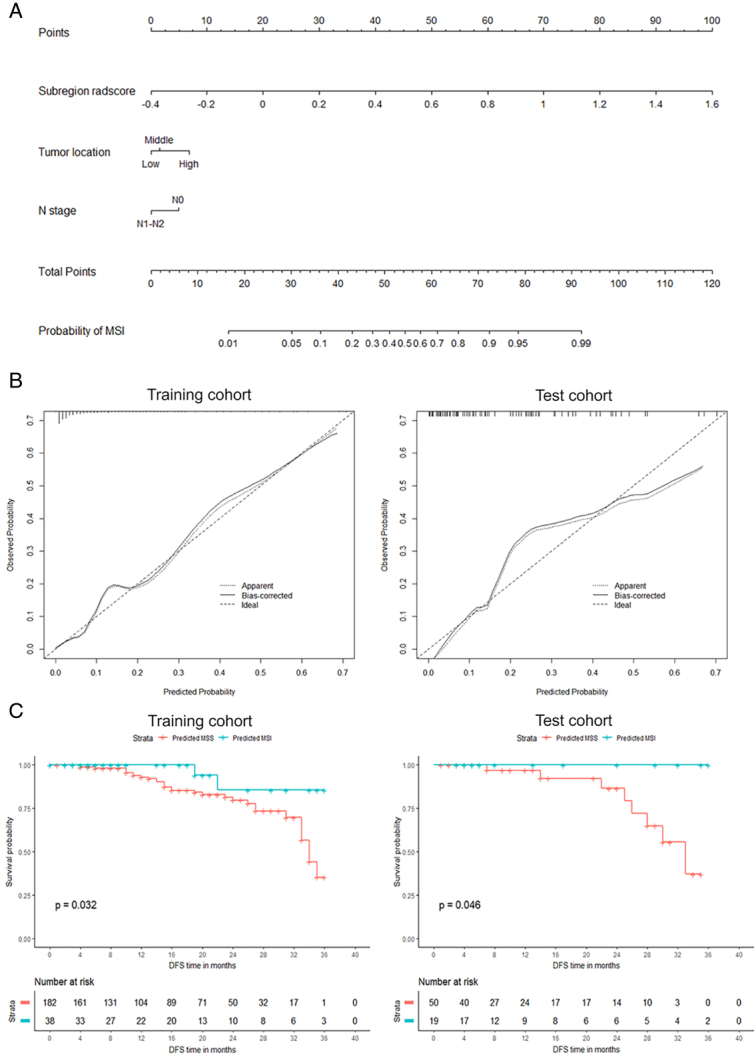
Explanation and application of subregion-clinicoradiological model (A)Nomogram for predicting microsatellite instability (MSI) of rectal cancer developed based on the training cohort (*n*=382). The nomogram takes into account the rad-score of the subregion radiomics model output, the tumor location, and the N stage. (B) Calibration curves of the subregion-clinicoradiological model for predicting MSI in the training cohort (left), external test cohort (right). The initial performance of the model without any corrections is represented by the dashed line. After addressing the observed bias, the model’s calibration is illustrated by the solid line. The diagonal line represents a scenario where the predicted probabilities perfectly match the observed probabilities. (C) Kaplan–Meier curves comparing 3-years DFS rate between predicted MSI and predicted MSS groups from the training cohort (left, 86 vs. 35%, P=.032), and external test cohort (right, 100 vs. 37%, *P*=.046).

The AUCs of the subregion radiomics model were superior to those of the clinicoradiological model in both training and external test cohorts (DeLong’s test, *P*<0.001 in the training cohort and *P*<0.01 in the external test cohort). However, no differences were observed among the AUCs of the subregion radiomics, subregion-clinicoradiological, and combined models (*P*>0.05 in both training and external test cohorts; Fig. 4A). In all cohorts, decision curve analysis showed no difference among the net benefits of the subregion radiomics, subregion-clinicoradiological, and combined models, whereas each of the three models demonstrated a greater net benefit than that of the clinicoradiological model (Fig. 4B). The AUC, accuracy, F1_score, sensitivity, and specificity of each model are presented in Table 2 and Figure 4C, and the subregion-clinicoradiological model preformed the optimal. Therefore, we selected the subregion-clinicoradiological model as the optimal model for nomogram construction (Fig. 5A). Across all patient cohorts, there were no observable differences between the calibration curves of the subregion-clinicoradiological model and the actual MSI status (Hosmer–Lemeshow test, *P*=0.64 in the training cohort and *P*=0.21 in the external test cohort) (Fig. 5B).

**Table 2:** Prediction performance of different models.

Cohort	Model	AUC (95% CI)	F1_score(95% CI)	Accuracy (95% CI)	Sensitivity (95% CI)	Specificity (95% CI)
Training cohort	Clinicoradiological	0.59 (0.49–0.69)	0.20 (0.14–0.29)	0.77 (0.44–0.82)	0.32 (0.18–0.81)	0.82 (0.42–0.86)
	Subregion radiomics	0.86 (0.79–0.93)	0.40 (0.33–0.54)	0.76 (0.73–0.90)	0.88 (0.68–0.96)	0.75 (0.72–0.91)
	Classical radiomics	0.72 (0.63–0.82)	0.30 (0.23–0.43)	0.73 (0.66–0.87)	0.68 (0.48–0.84)	0.73 (0.64–0.91)
	Subregion- clinicoradiological	0.87 (0.80–0.93)	0.47 (0.34–0.57)	0.84 (0.75–0.88)	0.82 (0.71–0.95)	0.84 (0.74–0.89)
	Combined model	0.86 (0.79–0.93)	0.40 (0.31–0.54)	0.78 (0.68–0.89)	0.82 (0.72–0.97)	0.78 (0.66–0.90)
External test cohort	Clinicoradiological	0.62 (0.48–0.75)	0.38 (0.14–0.52)	0.57 (0.52–0.86)	0.63 (0.10–0.87)	0.55 (0.48–0.99)
	Subregion radiomics	0.83 (0.74–0.92)	0.59 (0.42–0.74)	0.77 (0.55–0.87)	0.79 (0.64–1.00)	0.77 (0.44–0.88)
	Classical radiomics	0.73 (0.60–0.85)	0.47 (0.33–0.67)	0.59 (0.49–0.84)	0.89 (0.49–1.00)	0.51 (0.38–0.91)
	Subregion- clinicoradiological	0.85 (0.78–0.93)	0.63 (0.45–0.79)	0.80 (0.65–0.89)	0.84 (0.80–1.00)	0.78 (0.55–0.88)
	Combined model	0.86 (0.78–0.93)	0.63 (0.48–0.77)	0.80 (0.68–0.88)	0.84 (0.78–1.00)	0.78 (0.59–0.89)

AUC, area under the receiver operating characteristic curve.

### Prognostic analysis of the optimal model

To assess the prognostic stratification value of the subregion-clinicoradiological model, patients were stratified into predicted MSS and MSI groups using a cutoff value (0.145) that maximized the Youden index. Patients predicted to have MSI had a higher 3-year DFS rate (86% in the training cohort and 100% in the external test cohort) than those predicted to have MSS (35% in the training cohort and 37% in the external test cohort; log-rank test, *P*=0.032 and *P*=0.046, respectively). The survival curves are shown in Figure 5C.

## Discussion

In this study, we extracted multiparametric MRI subregion radiomic features to establish a novel model that could identify subregions in tumors that had the highest correlation with MSI. The model exhibited high diagnostic accuracy in predicting MSI and demonstrated significant robustness based on the data from two independent centers. When combined with clinicoradiological variables, it demonstrated the maximum performance. Furthermore, patients predicted to have MSI by the subregion-clinicoradiological model had a better prognosis than those predicted to have MSS.

The FCM and Bayesian Gaussian mixed models, which have complementary effects, were used for clustering in this study. Unlike traditional hard clustering methods, such as k-means clustering, FCM is a type of soft clustering. It introduces the concept of membership values, represented as a range of values from zero to one, to determine the cluster to which a data point belongs^37,38^. This approach appears to be more effective when the data become more difficult to cluster^39^. This method was particularly suitable for the initial clustering of multiparametric MRI images in our study. However, FCM has the limitation of a fixed number of clusters that do not align with the characteristics of tumor spatial heterogeneity^40^. To address this limitation, the Bayesian Gaussian mixed model, which can determine the optimal number of clusters without bias^41^, was employed in the second round of clustering. Although radiomics can offer additional information for clustering^28^, the number of variables used for clustering does not have a linear relationship with clustering accuracy^42^. PCA was employed to decrease the dimensionality of the data prior to the second round of clustering, and the information retained after dimensionality reduction accounted for 0.85 of the original information. This was performed to remove features that were not conducive to clustering and to enhance the accuracy and robustness of clustering^42^.

MRI-based multiparametric radiomics models have been shown to performs better than MRI-based single-parameter radiomics models, and the logistic regression algorithm is the most promising algorithm. Li *et al*.^18^ utilized the original radiomics features derived from multiparametric and single-parameter MRI for model development, and the model based on multiparametric images (AUC of 0.78 in the external test cohort) outperformed the models based on single sequence images (AUCs ranging from 0.67 to 0.77 in the external test cohort). Zhang *et al*.^19^ extracted the original, LoG, and wavelet radiomic features from multiparametric MRI and employed five machine-learning algorithms to construct models. They selected the logistic regression model as the optimal model, which achieved an AUC of 0.74 in the internal test cohort. Based on these findings, we constructed a subregion radiomics model based on multiparametric MRI using the logistic regression algorithm. Our model exhibited good performance (AUC of 0.83 in the external test cohort), surpassing those from previous studies and that of our classical radiomics model (AUC of 0.73 in the external test cohort). Our subregion radiomics model was built based on the features of subregion radiomics that were highly correlated with MSI. Therefore, for each patient, we could obtain and visualize the subregion locations that were highly correlated with the MSI. This may help overcome the problem of underestimating or overestimating the level of MMR protein expression detected at different biopsy locations owing to tumor heterogeneity^43^ and provide imaging guidance for biopsy locations in patients with RC.

In our study, there were no significant differences in the clinical characteristics between patients with MSI and those with MSS, which was similar to the findings of several previous studies^18,19^. However, in terms of radiological features, high RC vs. low RC [OR=3.02 (95% CI: 1.22–7.48)] showed a significant difference (*P*<0.05) between patients with MSI and MSS in multivariate analysis. This means that patients with RC closer to the colon are more likely to have MSI, which could be attributed to the higher tendency of colon cancer to be associated with MSI than that of RC^44^. Besides, N1–N2 vs. N0 [OR=0.39 (95% CI: 0.16–0.94)] was also significant, which may be attributed to the fact that RC with MSI status is usually associated with a better prognosis and a lower rate of lymph node metastasis^1^. However, the addition of these features improved the predictive ability of the subregion radiomics model only slightly. This suggests different biological characteristics between MSI and MSS RC.

This study had some limitations. First, this retrospective study may have led to selection bias. Although external validation was performed to improve reliability, further prospective analysis should be performed to reduce the impact of selection bias. Second, owing to the limited incidence of MSI in RC (10– 20%)^2^, there was only one external test center for this study. Hence, more external test centers are needed to validate the results of our study. Third, manual segmentation can be influenced by subjective evaluations, which may not be appropriate for handling a large sample of data. Therefore, developing an algorithm suitable for automatic segmentation is important. Lastly, we were unable to obtain the exact location of the pathological section in the whole tumor because of the retrospective nature of the study; therefore, the subregions associated with MSI that we obtained have not been further validated, and a prospective study will be conducted in the future.

## Conclusions

We developed and validated a subregion radiomics model based on multiparametric MRI to assess its high-risk subregions and preoperatively predict patients’ MSI status. When combined with clinicoradiological variables, the subregion model performed the optimal and stratified DFS successfully. The proposed model may facilitate clinical decisions regarding personalized treatment for patients with RC and positioning for biopsies based on images.

## Ethical approval

Ethics Committee of Shunde Hospital of Southern Medical University (research ethics 20201124).

## Consent

Written informed consent was obtained from the patient for publication of this case report and accompanying images. A copy of the written consent is available for review by the Editor-in-Chief of this journal on request.

## Sources of fundings

This research was supported by the traditional Chinese medicine research project of Guangdong (20241312); the Guangdong medical science and technology research fund (A2023204); the characteristic innovation projects of universities in Guangdong province (2023KTSCX022) Foshan Medical Imaging Artificial Intelligence Engineering Technology Application Research Center.

## Author contribution

Z.C., Z.X., Z.L., H.Z., and Q.H.: were responsible for the overall study design; Z.C., Z.X., Y.C., R.Z., X.C., D.L., C.L., X.L., W.L., C.Z., and X.G.: supervised the data collection; Z.C., Z.X., Y.C., and X.C.: performed data analysis; Z.C., Z.X., Y.C., and R.Z.: completed manuscript drafting; B.G., H.C., F.O., X.C., Z.L., H.Z., and Q.H.: were responsible for manuscript editing. All authors read, discussed, and approved the final version of the manuscript.

## Conflicts of interest statement

The authors declare no conflicts of interest.

## Research registration unique identifying number (UIN)

researchregistry9895, https://researchregistry.knack.com/researchregistry#home/registrationdetails/659d40861a4c34002909d610/.

## Guarantor

All the authors took responsibility of the final manuscript and approved it for publication.

## Data availability statement

The data where our results derived from were from Shunde Hospital, Southern Medical University and The First People’s Hospital of Foshan. The original data were not publicly available and could only be shared with the permission of the ethics committee of Shunde Hospital.

## Provenance and peer review

Not commissioned, externally peer-reviewed.

## Supplementary Material

SUPPLEMENTARY MATERIAL
