# Supplementary material for: Multiparametric MRI subregion radiomics for preoperative assessment of high-risk subregions in microsatellite instability of rectal cancer patients: a multicenter study
Source: Int J Surg. 2024 Mar 18;110(7):4310–9. doi: 10.1097/JS9.0000000000001335 (PMC11254239; doi:10.1097/JS9.0000000000001335)
Supplement: SUPPLEMENTARY MATERIAL [file js9-110-4310-s002.docx]

**Contents**

**Supplementary Method**

The rad-score formula of each subregion

**Supplementary Tables**

Supplementary Table 1. MRI scanning parameters

Supplementary Table 2. Consistency analysis of radiological features in different cohorts

Supplementary Table 3. Univariable and multivariable logistic regression analysis for the association between MSI and MSS patient characteristics

Supplementary Table 4. Complete radiomic features selection process

**Supplementary Figures**

Supplementary Figure 1. Forest plot of selected subregion and classical features

Supplementary Figure 2. Features importance plot based on the features coefficients of subregion radiomics model.

**Supplementary Methods**

The rad-score formula of each subregion

Rad-score = 0.0985 - (0.0363×original_glszm_SmallAreaHighGrayLevelEmphasis_T1WI) - (0.00294×original_firstorder_RootMeanSquared_CE-T1WI) + (0.0105×original_gldm_DependenceNonUniformityNormalized_DWI) + (0.0170×original_gldm_LargeDependenceHighGrayLevelEmphasis_T2WI) + (0.0409×original_glszm_SizeZoneNonUniformityNormalized_DWI) - (0.0461×original_glcm_InverseVariance_DWI) + (0.183×original_gldm_DependenceNonUniformity_T1WI)

**Supplementary Table 1: MRI scanning parameters**

| Institution | Scanner | Sequence | TR/TE(ms) | FOV(mm) | Matrix | Section Thickness(mm) | Section Gap(mm) | Slices | Flip Angle |
| --- | --- | --- | --- | --- | --- | --- | --- | --- | --- |
| Center A | DISCOVERY MR750x(3.0T) | T1WI | 597/9.7 | 340×340 | 320×256 | 6 | 1 | 32 | NA |
|  |  | T2WI | 4361.5/140.82 | 320×320 | 320×224 | 6 | 1 | 32 | NA |
|  |  | DWI | 4900/69.35 | 320×256 | 128×128 | 6 | 1 | 32 | NA |
|  |  | CE-T1WI | 4.49/1.745 | 360×324 | 256×256 | 4.5 | -2.5 | 128 | 12 |
|  | Signa HDxt(3.0T) | T1WI | 408.34/10.14 | 310×310 | 228×224 | 6 | 1 | 30 | NA |
|  |  | T2WI | 3550/80.19 | 350×350 | 228×224 | 6 | 1.5 | 30 | NA |
|  |  | DWI | 5337.5/73.30 | 380×380 | 128×128 | 6 | 1 | 30 | NA |
|  |  | CE-T1WI | 3.53/1.68 | 400×360 | 256×192 | 5 | -2.5 | 92 | 15 |
|  | OPTIMA MR360(1.5T) | T1WI | 405/13.22 | 410×328 | 288×224 | 6 | 2 | 36 | NA |
|  |  | T2WI | 4658.5/90.76 | 410×328 | 288×224 | 6 | 2 | 36 | NA |
|  |  | DWI | 5875/77.6 | 410×410 | 96×128 | 6 | 2 | 36 | NA |
|  |  | CE-T1WI | 6.28/3.13 | 420×378 | 288×192 | 5 | -2.4 | 115 | 12 |
| Center B | Skyra(Siemes, 3.0T) | T1WI | 804/9.3 | 280×280 | 384×307 | 3.0 | 0.3 | 35 | 132 |
|  |  | T2WI | 6200/89 | 240×240 | 384×384 | 3.0 | 0.3 | 35 | 150 |
|  |  | DWI | 6700/63 | 240×240 | 114×114 | 3.0 | 0.3 | 35 | NA |
|  |  | CE-T1WI | 781/9 | 280×280 | 320×272 | 3.0 | 0.3 | 35 | 132 |
|  | Lumina(Siemes, 3.0T) | T1WI | 804/9.3 | 280×280 | 384×307 | 3.0 | 0.3 | 35 | 132 |
|  |  | T2WI | 8980/96 | 240×240 | 352×299 | 3.0 | 0.3 | 35 | 150 |
|  |  | DWI | 7100/90 | 200×200 | 114×114 | 2.5 | 0.5 | 30 | NA |
|  |  | CE-T1WI | 781/9 | 280×280 | 320×272 | 3.0 | 0.3 | 35 | 132 |

**Supplementary Table 2: Consistency analysis of radiological features in different cohorts**

|  | **Training cohort(n=382)** | | | **Test cohort(n=93)** | | |
| --- | --- | --- | --- | --- | --- | --- |
| **Radiological characteristics** | **Radiologist A** | **Radiologist B** | **κ(P value)** | **Radiologist A** | **Radiologist B** | **κ(P value)** |
| **Tumor location** |  |  |  |  |  |  |
| Low | 169 | 164 | 0.955(<.001) | 48 | 49 | 0.981(<.001) |
| Middle | 138 | 142 |  | 39 | 38 |  |
| High | 75 | 76 |  | 6 | 6 |  |
| **T stage** |  |  | 0.873(<.001) |  |  | 0.807(<.001) |
| T1-T2 | 115 | 128 |  | 28 | 34 |  |
| T3-T4 | 267 | 254 |  | 65 | 59 |  |
| **N stage** |  |  | 0.770(<.001) |  |  | 0.837(<.001) |
| N0 | 252 | 257 |  | 59 | 60 |  |
| N1-N2 | 130 | 125 |  | 34 | 33 |  |

**Supplementary Table 3：Univariable and multivariable logistic regression analysis for the association between MSI and MSS patient characteristics**

| Characteristics | OR(univariable) | 95%CI | P. Value | OR (multivariable) | 95%CI | P. Value |
| --- | --- | --- | --- | --- | --- | --- |
| **Age** | 1.00 | 0.97,1.03 | 0.840 |  |  |  |
| **Sex** |  |  |  |  |  |  |
| Male | reference |  |  |  |  |  |
| Female | 0.73 | 0.34,1.58 | 0.431 |  |  |  |
| **CEA** |  |  |  |  |  |  |
| ≤5ug/L | reference |  |  |  |  |  |
| >5ug/L | 1.01 | 0.50,2.05 | 0.986 |  |  |  |
| **CA19-9** |  |  |  |  |  |  |
| ≤37 U/ml | reference |  |  |  |  |  |
| >37 U/ml | 0.40 | 0.09,1.73 | 0.219 |  |  |  |
| **Tumor Location** |  |  |  |  |  |  |
| Low | reference |  |  | reference |  |  |
| Middle | 1.37 | 0.58,3.20 | 0.470 | 1.68 | 0.70,4.01 | 0.242 |
| High | 2.47 | 1.02,5.98 | 0.045 | 3.02 | 1.22,7.48 | 0.017 |
| **T stage** |  |  |  |  |  |  |
| T1-T2 | reference |  |  |  |  |  |
| T3-T4 | 1.22 | 0.55,2.70 | 0.629 |  |  |  |
| **N stage** |  |  |  |  |  |  |
| N0 | reference |  |  | reference |  |  |
| N1-N2 | 0.47 | 0.20,1.12 | 0.089 | 0.39 | 0.16,0.94 | 0.037 |

**Supplementary Table 4: Complete radiomic features selection process**

| Method | Subregion radiomic features | Classical radiomic features |
| --- | --- | --- |
| All features | original_shape:56 | original_shape:56 |
|  | original_firstorder:72 | original_firstorder:72 |
|  | original_glcm:92 | original_glcm:92 |
|  | original_glrlm:64 | original_glrlm:64 |
|  | original_glszm:64 | original_glszm:64 |
|  | original_gldm:56 | original_gldm:56 |
|  | original_ngtdm:20 | original_ngtdm:20 |
| Assessment of inter- and intra-observer reliability  using intraclass correlation coefficients(ICCs) | original_shape:48 | original_shape:48 |
|  | original_firstorder:65 | original_firstorder:65 |
|  | original_glcm:65 | original_glcm:65 |
|  | original_glrlm:45 | original_glrlm:45 |
|  | original_glszm:50 | original_glszm:50 |
|  | original_gldm:39 | original_gldm:39 |
|  | original_ngtdm:16 | original_ngtdm:16 |
| Univariate logistic regression | original_shape:44 | original_shape:40 |
|  | original_firstorder:46 | original_firstorder:29 |
|  | original_glcm:44 | original_glcm:28 |
|  | original_glrlm:37 | original_glrlm:24 |
|  | original_glszm:38 | original_glszm:26 |
|  | original_gldm:36 | original_gldm:23 |
|  | original_ngtdm:15 | original_ngtdm:14 |
| Pearson's correlation analysis | original_shape:4 | original_shape:2 |
|  | original_firstorder:10 | original_firstorder:8 |
|  | original_glcm:14 | original_glcm:7 |
|  | original_glrlm:3 | original_glrlm:6 |
|  | original_glszm:7 | original_glszm:6 |
|  | original_gldm:16 | original_gldm:7 |
|  | original_ngtdm:7 | original_ngtdm:5 |
| The least absolute shrinkage and selection operator (LASSO) | original_shape:1 | original_shape:2 |
|  | original_firstorder:5 | original_firstorder:4 |
|  | original_glcm:7 | original_glcm:3 |
|  | original_glrlm:2 | original_glrlm:4 |
|  | original_glszm:3 | original_glszm:3 |
|  | original_gldm:6 | original_gldm:1 |
|  | original_ngtdm:2 |  |
| stepwise logistic regression | original_firstorder:1 | original_shape:1 |
|  | original_glcm:1 | original_firstorder:1 |
|  | original_glszm:2 | original_glcm:1 |
|  | original_gldm:3 | original_glszm:2 |
|  |  | original_gldm:1 |


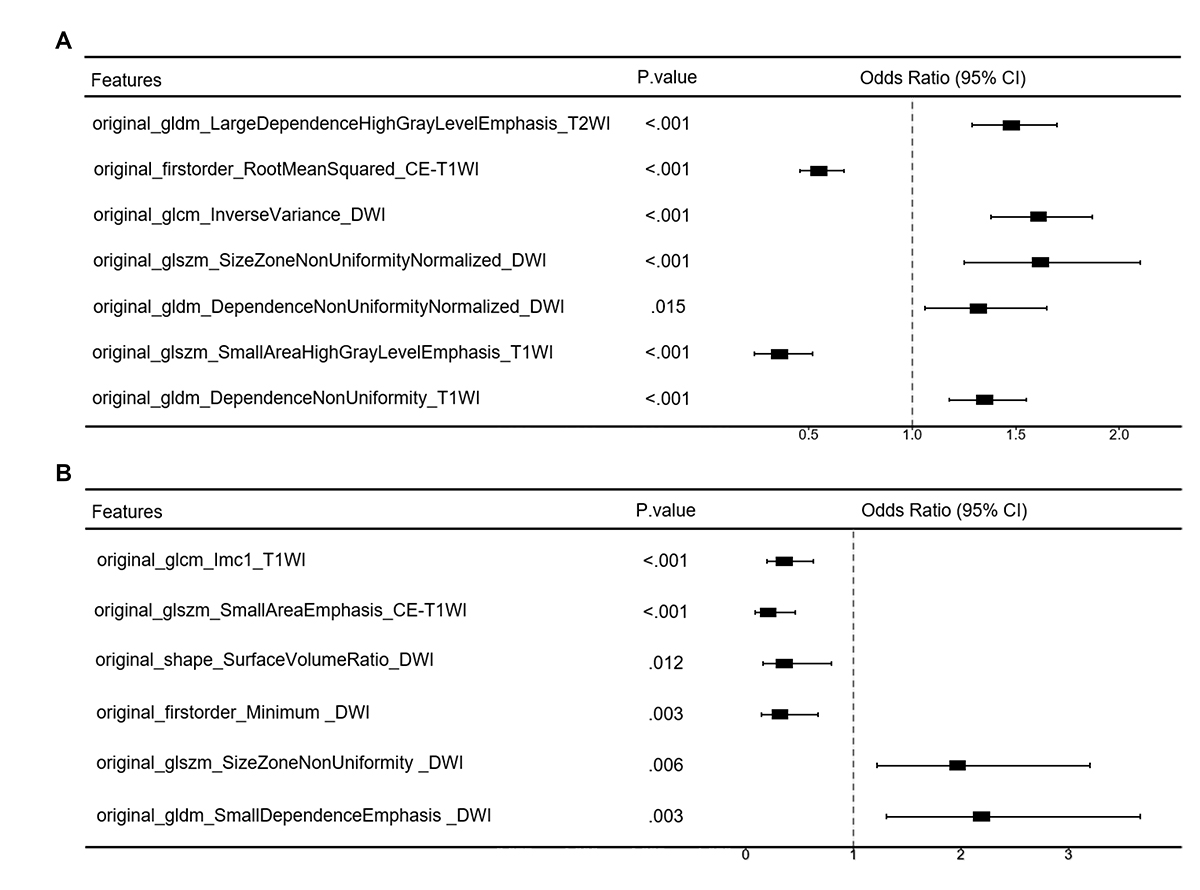


**Supplementary Figure 1: Forest plot of** **selected subregion (A) and classical (B) features. Their p values are described in the table, and the odds ratios are plotted as line segments with black square.**


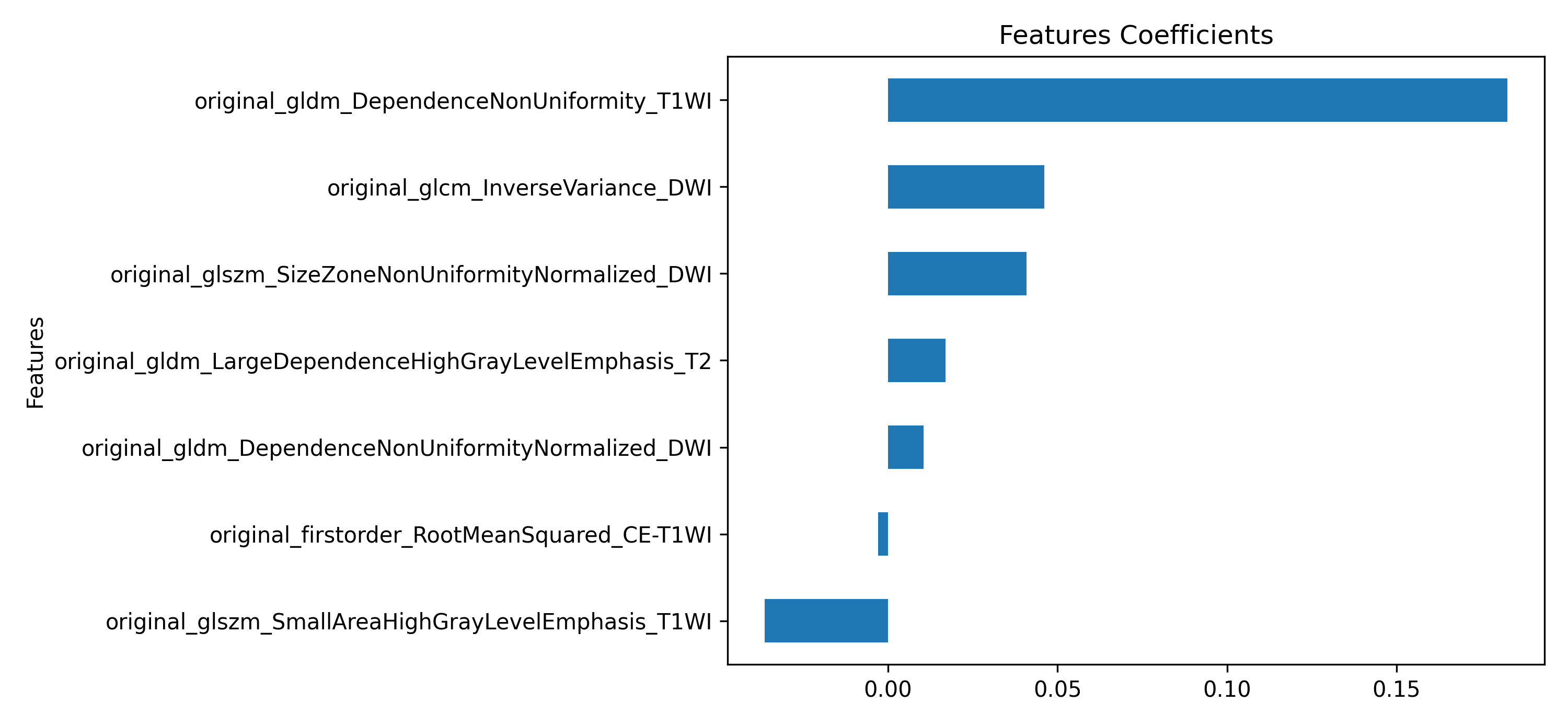


**Supplementary Figure 2: Features importance plot based on the features coefficients of subregion radiomics model.**
